# The Receptor for Advanced Glycation End Products (RAGE) Is Associated with Persistent Atrial Fibrillation

**DOI:** 10.1371/journal.pone.0161715

**Published:** 2016-09-14

**Authors:** Terase F. Lancefield, Sheila K. Patel, Melanie Freeman, Elena Velkoska, Bryan Wai, Piyush M. Srivastava, Mark Horrigan, Omar Farouque, Louise M. Burrell

**Affiliations:** 1 Department of Medicine, University of Melbourne, Austin Health, Heidelberg, Victoria, Australia; 2 Department of Cardiology, Austin Health, Heidelberg, Victoria, Australia; 3 Department of Cardiology, Box Hill Hospital, Box Hill, Victoria, Australia; University of Pittsburgh, UNITED STATES

## Abstract

**Objective:**

Upregulation of the receptor for advanced glycation end products (RAGE) has been proposed as a pathophysiological mechanism underlying the development of atrial fibrillation (AF). We sought to investigate if soluble RAGE levels are associated with AF in Caucasian patients.

**Methods:**

Patients (n = 587) were prospectively recruited and serum levels of soluble RAGE (sRAGE) and endogenous secretory RAGE (esRAGE) measured. The patients included 527 with sinus rhythm, 32 with persistent AF (duration >7 days, n = 32) and 28 with paroxysmal AF (duration <7 days, n = 28).

**Results:**

Patients with AF were older and had a greater prevalence of heart failure than patients in sinus rhythm. Circulating RAGE levels were higher in patients with persistent AF [median sRAGE 1190 (724–2041) pg/ml and median esRAGE 452 (288–932) pg/ml] compared with paroxysmal AF [sRAGE 799 (583–1033) pg/ml and esRAGE 279 (201–433) pg/ml, p ≤ 0.01] or sinus rhythm [sRAGE 782 (576–1039) pg/ml and esRAGE 289 (192–412) pg/ml, p < 0.001]. In multivariable logistic regression analysis, independent predictors of persistent AF were age, heart failure, sRAGE [odds ratio 1.1 per 100 pg/ml, 95% confidence interval (CI) 1.0–1.1, p = 0.001] and esRAGE [odds ratio 1.3 per 100 pg/ml, 95% CI 1.1–1.4, p < 0.001]. Heart failure and age were the only independent predictors of paroxysmal AF. In AF patients, sRAGE [odds ratio 1.1 per 100 pg/ml, 95% CI 1.1–1.2, p = 0.007] and esRAGE [odds ratio 1.3 per 100 pg/ml, 95% CI 1.0–1.5, p = 0.017] independently predicted persistent compared with paroxysmal AF.

**Conclusions:**

Soluble RAGE is elevated in Caucasian patients with AF, and both sRAGE and esRAGE predict the presence of persistent AF.

## Introduction

Atrial fibrillation (AF) is the most common sustained cardiac arrhythmia and is found in 1% of the population, with a prevalence that increases as the population ages. [1] The precise molecular mechanisms involved in atrial remodeling remain incompletely understood, but it is clear that pro-fibrotic and pro-inflammatory pathways increase the vulnerability to AF. [2, 3] One such pathway that occurs with aging involves the non-enzymatic binding of proteins and lipids leading to the formation of advanced glycation end-products (AGEs). [4, 5] AGEs may play a role in the pathophysiology of AF through their interaction with the cellular receptor for AGE (RAGE), [6] which causes inflammation and fibrosis via sustained activation of nuclear factor-kappaB [7] and upregulation of cytokines and adhesion molecules. [8] In addition to its presence on cells, isoforms of RAGE (sRAGE) exist in the circulation including full-length cell-surface RAGE which results from proteolytic cleavage (cleaved RAGE), and endogenous secretory RAGE (esRAGE), a splice variant that binds RAGE ligands but lacks the cytosolic tail critical for signal transduction. [9]

Although there is some evidence that circulating forms of soluble RAGE may have clinical utility as biomarkers of AF, [10–12] the 3 studies to date have produced varying results. This may be due to the biomarker measured (sRAGE or esRAGE), the type of AF patient studied (paroxysmal or persistent AF), or the ethnicity of the patient (Chinese, [10, 12] European [11]). This study aimed to clarify the possible association between levels of soluble RAGE and AF through the measurement of both sRAGE and esRAGE in Caucasian patients with sinus rhythm (SR), persistent AF and paroxysmal AF. We hypothesized that RAGE signaling plays a role in the maintenance of AF, and therefore that serum levels of sRAGE and esRAGE may differ according to whether AF was persistent or paroxysmal.

## Materials and Methods

### Study sample

We prospectively enrolled patients presenting for cardiac investigations between 2008 and 2011 at Austin Health, Melbourne, Australia. For the purpose of this study, patients of non-Caucasian ethnicity and those with a paced rhythm were excluded. The research was carried out according to the Declaration of Helsinki (2000) of the World Medical Association, and was approved by the Human Research Ethics Committee at Austin Health, Melbourne. All participants provided written consent.

### Clinical measurements and definitions

A standardized medical questionnaire was completed and verified with the hospital medical record. Blood pressure was measured in the supine position and anthropometric measurements were taken. A 12-lead ECG was recorded to determine heart rate and rhythm. Paroxysmal AF was defined as having paroxysms of AF that terminated within 7 days of onset. [13] All patients with a documented history of AF who were in SR at the time of recruitment were considered to have paroxysmal AF. Persistent AF was defined as AF lasting >7 days and included patients with long-standing persistent and permanent AF. Diabetes was diagnosed based on a documented history, treatment with anti-diabetic medication or when the fasting blood glucose was >7 mmol/L. Hypertension was diagnosed as present if previously diagnosed by a physician and/or there was current use of anti-hypertensive medication. Dyslipidemia was defined as present if previously diagnosed by a physician and/or there was current use of lipid lowering agents. Cigarette smoking was defined as smoking within the preceding 12 months. Obesity was diagnosed when the body mass index was >30 kg/m^2^. Renal impairment was diagnosed when the estimated glomerular filtration rate (eGFR) was <60 ml/min/1.73m^2^. Left ventricular (LV) ejection fraction was calculated with the modified Simpson method or quantitative LV angiography. LV dysfunction was defined as a LV ejection fraction <50%. Heart failure was diagnosed when there was evidence of previous dyspnea, fluid retention or low cardiac output secondary to cardiac dysfunction, or documented rales, jugular venous distension or pulmonary edema prior to the current admission. Valvular heart disease was diagnosed when left sided valve dysfunction was severe on recent echocardiography. Previous myocardial infarction was diagnosed when there was evidence of a typical rise and gradual fall of troponin I associated with either ischemic symptoms or ECG changes according to standard criteria. [14]

### Biochemical Analyses

Blood samples were collected at enrollment and serum was obtained by centrifuging blood at 3000 rpm at 4°C for 10 minutes. Specimens were stored at –80°C. Plasma electrolytes were measured on a Hitachi 911 automatic analyzer (Roche Diagnostics, Mannheim, Germany). The eGFR was calculated using the four-component abbreviated Modification of Diet in Renal Disease equation. Glycated hemoglobin (HbA1c) was measured by automated high-performance liquid chromatography (Biorad Diamat, CA, USA), and fasting lipids by enzymatic colorimetry. Total cholesterol was measured by enzymatic colorimetric methods and low-density lipoprotein cholesterol was calculated using the Friedewald equation. Serum C-reactive protein levels were measured using the Synchron LX® system on a Beckman Coulter analyser.

Serum sRAGE (R&D Systems, Minneapolis, MN) and esRAGE (B-Bridge International, Cupertino, CA) were measured using a sandwich ELISA according to the manufacturer’s instructions. The limit of detection was 4.1 pg/ml and 25 pg/ml respectively. The intra-assay and inter-assay coefficients of variation values for sRAGE were 6.0% and 7.2% respectively and for esRAGE were 1.5% and 10.0% respectively. Samples were run in duplicate and the mean values used for analysis.

### Statistical analyses

Analysis was performed using SPSS version 20 (SPSS Inc., Chicago, IL, USA). Categorical variables are expressed as n (%) and assessed using Fisher’s exact tests. Continuous variables are expressed as mean ± standard deviation and assessed using one-way between-group analysis of variance. Post-hoc comparisons were conducted using Fisher’s least significant difference. Serum RAGE and C-reactive protein were non-normally distributed and were compared using the Kruskal-Wallis test. Median values and 25^th^-75^th^ percentiles are presented in the results. Subgroup comparisons were conducted using the Mann-Whitney U test. Spearman’s correlation coefficient (r_s_) was used to determine the relationship between sRAGE and esRAGE. As diabetes accelerates glycation and is an independent risk factor for AF, [4, 15, 16] we specifically examined whether diabetes influenced the relationship between AF and serum RAGE using generalized linear model analysis, with serum RAGE as the dependent variable and persistent AF and diabetes as explanatory variables. The interaction term for AF and diabetes was examined in this model. Multivariable logistic regression analysis was performed to determine independent predictors of paroxysmal and persistent AF compared with SR using clinical variables potentially associated with AF, with a p-value <0.1 in univariable analysis. Variables considered included age, gender, obesity, hypertension, diabetes, smoking, renal impairment, myocardial infarction, heart failure, valvular heart disease, insulin use, angiotensin converting enzyme inhibitor or angiotensin II receptor blocker use, statin use, systolic blood pressure, low density lipoprotein cholesterol, HbA1_c_, C-reactive protein and circulating sRAGE and esRAGE. The same variables were considered when predicting persistent versus paroxysmal AF in addition to antiarrhythmic use. Serum sRAGE and esRAGE were highly correlated and were analyzed in separate models to avoid issues with multicollinearity. Two-tailed p-values <0.05 were considered significant.

## Results

### Clinical and biochemical characteristics according to heart rhythm

We recruited 681 patients and excluded 87 patients of non-Caucasian ethnicity and 7 patients with a cardiac pacemaker. In the remaining 587 patients, 60 (10.2%) had AF, of whom 32 (5.5%) had persistent AF and 28 (4.8%) had paroxysmal AF. The clinical and biochemical characteristics of the study population are presented in Table 1. Patients with AF (either persistent or paroxysmal) were older and more likely to have heart failure than patients in SR (both p ≤ 0.001). Prevalence of diabetes and other cardiac risk factors including hypertension, dyslipidemia, obesity and smoking were similar across the groups. Patients with persistent AF were more likely to have renal impairment and a prior stroke and were less likely to be taking statins than patients in SR (all p < 0.05). Warfarin use was significantly higher in patients with AF compared with SR (p < 0.001), and was greatest in those with persistent compared with paroxysmal AF. No patients were taking novel oral anticoagulant agents. Antiarrhythmic agents were commonly used in patients with paroxysmal and persistent AF. Over two-thirds of the total cohort were taking renin-angiotensin system blockers, with similar use in all 3 groups. Heart rate was higher in patients with persistent AF compared with the other groups while blood pressure was similar. Estimated GFR was lower in patients with persistent AF compared with SR (p = 0.009) and C-reactive protein was higher in patients with paroxysmal AF compared with SR (p = 0.03); levels of both markers were similar in patients with paroxysmal and persistent AF. Low density lipoprotein cholesterol, high-density lipoprotein cholesterol and triglycerides levels were similar in all 3 groups (all p > 0.05). Glycemic control was similar in diabetic patients with SR (HbA1c = 7.4 ± 1.2%), paroxysmal AF (HbA1c = 7.8 ± 1.6%) and persistent AF (HbA1c = 7.3 ± 1.2%), p = 0.47.

**Table 1:** Clinical characteristics of study patients.

	Sinus Rhythm	Paroxysmal AF	Persistent AF	P Value
	(n = 527)	(n = 28)	(n = 32)	
Age, years	64 ± 11	70 ± 10†	70 ± 10†	**0.001**
Female	178 (34%)	10 (36%)	9 (28%)	0.80
Obesity	237 (45%)	16 (57%)	15 (47%)	0.45
Diabetes mellitus	294 (56%)	16 (57%)	16 (50%)	0.82
Hypertension	426 (80%)	25 (89%)	27 (84%)	0.50
Dyslipidemia	434 (82%)	20 (71%)	22 (69%)	0.06
Myocardial infarction	111 (21%)	10 (36%)	5 (16%)	0.13
Heart failure	29 (6%)	10 (36%)†	12 (38%)†	**<0.001**
Valvular heart disease	28 (5%)	4 (14%)	4 (13%)	**0.03**
Renal impairment	110 (21%)	10 (36%)	12 (38%)†	**0.02**
Cigarette smoking	75 (14%)	3 (11%)	1 (3%)	0.19
Stroke	20 (4%)	2 (7%)	5 (16%)†	**0.01**
**Medications**				
Warfarin	10 (2%)	5 (18%)†	24 (75%)†‡	**<0.001**
Antiplatelet	392 (74%)	20 (71%)	10 (31%)†‡	**<0.001**
Beta-blocker	245 (47%)	18 (64%)	22 (69%)†	**0.01**
Antiarrhythmic	3 (0.6%)	11 (39%)†	5 (16%)†‡	**<0.001**
ACEi or ARB	366 (69%)	24 (86%)	21 (66%)	0.15
Diuretic	183 (35%)	17 (61%)†	21 (66%)†	**<0.001**
Statin	387 (73%)	20 (71%)	17 (53%)†	0.05
Insulin	145 (28%)	4 (14%)	6 (19%)	0.20
**Physical Parameters**				
Heart rate, beats/min	67 ± 13	65 ± 14	79 ± 21†‡	**<0.001**
Systolic BP, mmHg	133 ± 23	139 ± 21	132 ± 23	0.43
LV dysfunction	77 (15%)	7 (26%)	9 (29%)	**0.05**
**Biochemical Parameters**				
Renal impairment	80 ± 28	74±29	67±24†	**0.02**
LDL cholesterol, mmol/L	2.4 ± 1.0	2.2 ± 1.0	2.2 ± 1.0	0.36
C-reactive protein, mg/L	2.7 (1.2, 5.3)	7.3 (2.0, 11.2)†	4.6 (1.3, 8.3)	**0.03**
Serum sRAGE, pg/ml	782 (576, 1039)	799 (583, 1033)	1190 (724, 2041)†‡	**0.001**
Serum esRAGE, pg/ml	289 (192, 412)	279 (201, 433)	452 (288, 932)†‡	**0.001**

Subgroup analysis:

† p <0.05 vs. sinus rhythm

‡ p <0.05 vs. paroxysmal AF.

Values are total numbers and proportion (%), mean ± SD, or median (25, 75^th^ percentile). ACEi = angiotensin converting enzyme inhibitor; AF = atrial fibrillation; ARB = angiotensin II receptor blocker; BP = blood pressure; LDL = low-density lipoprotein; LV = left ventricular; RAGE = receptor for advanced glycation end products.

### Serum RAGE levels

**Fig 1:**
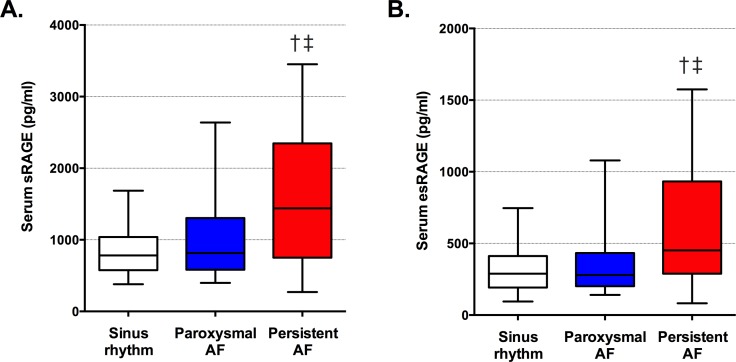
**Serum sRAGE (A) and esRAGE (B) levels were significantly elevated in patients with persistent AF.** Median and interquartile ranges are shown. Error bars indicate 5^th^ to 95^th^ percentiles. † Sinus rhythm vs. persistent AF, both p < 0.001; ‡ paroxysmal AF vs. persistent AF, both p ≤ 0.01.

Serum RAGE levels in the study cohort are shown (S1 File). Serum sRAGE and esRAGE were highly correlated in patients with SR (r_s_ = 0.87, p<0.001), paroxysmal AF (r_s_ = 0.89, p<0.001) and persistent AF (r_s_ = 0.94, p<0.001). Median serum sRAGE and esRAGE levels were significantly higher in patients with persistent AF compared with SR (both p<0.001) and paroxysmal AF (both p≤0.01) (Table 1 and Fig 1).

**Fig 2:**
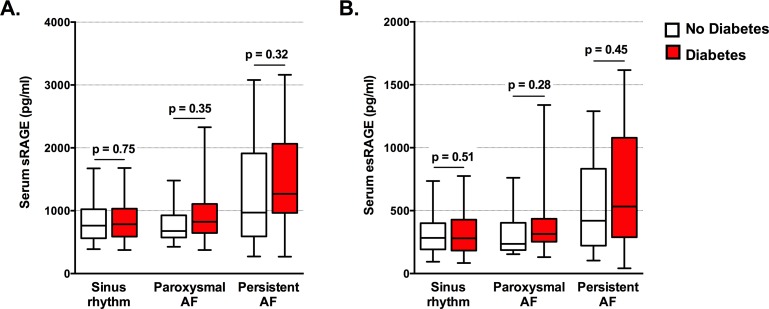
**Serum sRAGE (A) and esRAGE (B) levels did not differ according to the presence of diabetes.** Median and interquartile ranges are shown. Error bars indicate 5^th^ to 95^th^ percentiles. P values are shown.

We also examined sRAGE and esRAGE levels according to diabetes status, given that diabetes is a condition of accelerated glycation (Fig 2). There were 326 patients with diabetes of whom 294 were in SR, 16 had paroxysmal AF and 16 had persistent AF. Serum sRAGE and esRAGE levels did not differ according to the presence or not of diabetes. Furthermore, diabetes did not influence the relationship between persistent AF and serum RAGE, with no interaction between persistent AF and diabetes for sRAGE (p = 0.20) or esRAGE (p = 0.24).

### Predictors of atrial fibrillation compared with sinus rhythm

The independent predictors of persistent AF compared with SR are shown in Table 2. Heart failure, age, serum sRAGE (model 1) and serum esRAGE (model 2) independently predicted persistent AF, while C-reactive protein and renal impairment did not. Heart failure was the strongest predictor of persistent AF, but even after exclusion of patients with heart failure, both sRAGE [OR 1.1 per 100 pg/ml, 95% CI 1.1, 1.2, p < 0.001] and esRAGE [OR 1.3 per 100 pg/ml, 95% CI 1.2, 1.5, p < 0.001] remained significantly associated with persistent AF. The independent predictors of paroxysmal AF compared with SR were heart failure [OR 9.1, 95%CI 3.7, 22.5, p<0.001] and age [OR 1.6 per decade, 95%CI 1.1, 2.5, p = 0.028], while C-reactive protein did not reach statistical significance.

**Table 2:** Multivariable logistic regression analysis demonstrating predictors of persistent atrial fibrillation compared with sinus rhythm.

Variables	Model 1: sRAGE		Model 2: esRAGE	
	OR (95% CI)	P Value	OR (95% CI)	P Value
Age (per decade)	1.63 (1.10, 2.43)	**0.02**	1.56 (1.05, 2.31)	**0.03**
Heart failure	7.12 (2.92, 17.40)	**<0.001**	6.15 (2.45, 15.45)	**<0.001**
Renal impairment	0.78 (0.32, 1.89)	0.58	0.72 (0.29, 1.79)	0.72
C-reactive protein (per 1mg/L)	1.01 (1.00, 1.02)	0.09	1.01 (1.00, 1.02)	0.07
Serum RAGE (per 100 pg/ml)	1.08 (1.03, 1.13)	**0.001**	1.25 (1.11, 1.40)	**<0.001**

CI = confidence intervals; OR = odds ratio; RAGE = receptor for advanced glycation end products

### Predictors of persistent compared with paroxysmal atrial fibrillation

The independent predictors of persistent compared with paroxysmal AF are shown in Table 3. Both serum sRAGE (OR 1.3 per 100 pg/ml, 95%CI 1.0, 1.2, p = 0.007) and esRAGE (1.26 95%CI 1.04, 1.51, p = 0.017) were associated with an increased risk of persistent compared with paroxysmal AF. Antiarrhythmic use and renin-angiotensin system blockers were not statistically significant.

**Table 3:** Multivariable logistic regression analysis demonstrating predictors of persistent compared with paroxysmal atrial fibrillation.

Variables	Model 1: sRAGE		Model 2: esRAGE	
	OR (95% CI)	p Value	OR (95% CI)	P Value
Antiarrhythmic agent	0.38 (0.10, 1.18)	0.16	0.16 (0.10, 1.48)	0.16
ACEi or ARB	0.31 (0.08, 1.25)	0.10	0.30 (0.08, 1.17)	0.08
Serum RAGE (per 100 pg/ml)	1.13 (1.03, 1.24)	**0.007**	1.26 (1.04, 1.51)	**0.02**

ACEi = angiotensin converting enzyme inhibitor; ARB = angiotensin II receptor blocker; CI = confidence intervals; OR = odds ratio; RAGE = receptor for advanced glycation end products

## Discussion

This is the first study in Caucasian patients to measure serum levels of both sRAGE and esRAGE in patients with SR, persistent AF and paroxysmal AF. Serum sRAGE and esRAGE were significantly higher in patients with persistent AF and predicted the presence of persistent AF compared with SR. An important finding was that both elevated serum sRAGE and esRAGE independently predicted persistent compared with paroxysmal AF.

Our results confirm and extend the work of others that increased serum sRAGE levels predict persistent AF compared with SR [10, 11] and persistent AF compared with paroxysmal AF. [10] By contrast to our finding that elevated esRAGE predicted persistent compared with paroxysmal AF, lower esRAGE was associated with AF in patients of Chinese ethnicity [10, 12] and Chinese patients with persistent AF had lower levels of esRAGE compared to patients with paroxysmal AF. [10] Although there have been limited studies examining RAGE in AF, soluble RAGE has been extensively studied in other forms of cardiovascular disease. The strong correlation in this study between serum sRAGE and esRAGE levels is in agreement with the work of Colhoun et al [17] who measured sRAGE and esRAGE in patients with diabetes (predominantly Caucasian), and reported that the 2 markers were highly correlated (r = 0.88), with elevations predicting incident coronary artery disease events. In Caucasian patients with end-stage renal failure, sRAGE and esRAGE were also highly correlated (r = 0.95). [18] By contrast, studies in patients of Chinese or Japanese ethnicity have reported that lower levels of esRAGE were associated with coronary artery disease severity, [19] increased carotid intima-media thickness [20] and heart failure [21], and an inverse association has been noted between esRAGE and sRAGE levels. [21]

Ethnic differences in serum sRAGE and esRAGE levels have been noted in multiple previous studies. [17, 22, 23] Interestingly, the G82S SS polymorphism of the RAGE gene within the ligand-binding domain of the receptor is associated with reduced serum esRAGE levels in Chinese patients and reduced serum sRAGE levels in Korean patients, yet this same polymorphism was not related to sRAGE or esRAGE levels in Caucasian patients. [18, 24, 25] In other studies, the G82S SS polymorphism is associated with an increased prevalence of coronary artery disease and inflammation in Chinese patients, an increased prevalence of rheumatoid arthritis in Caucasian patients and enhanced ligand binding and cytokine generation in tissue culture studies. [26, 27] Taken together with our own findings, we speculate that differences in ethnicity and gene polymorphisms could explain much of the discordant findings from previous studies of these soluble receptors. This raises the question of whether RAGE gene polymorphisms need to be considered when interpreting levels of these soluble receptors, particularly when multiple ethnic groups are being studied together.

### Clinical predictors of atrial fibrillation

Both heart failure and age were independently associated with AF. Although heart failure was the strongest predictor of persistent AF compared with SR, serum RAGE independently predicted AF in multivariable analysis. When heart failure patients were excluded from analysis, sRAGE and esRAGE remained significantly associated with persistent AF. These results are supported by the observations of Raposeiras-Roubin et al, [28] who noted a positive association between persistent AF and serum sRAGE in patients with chronic heart failure. Furthermore, although heart failure was a risk factor for developing AF in the present study, it did not differentiate paroxysmal from persistent AF, suggesting that other factors (possibly RAGE) may be of importance in the maintenance of AF. Glycation may be particularly relevant in elderly subjects, where the abundance of glycated protein is increased by virtue of age. [5]

Diabetes is associated with accelerated formation of AGEs, [4, 5] which is of interest as patients with diabetes share a similar risk factor profile to AF and diabetes independently contributes to AF development. [15, 16] However, we found no independent association between AF and diabetes. Our finding that serum RAGE levels were similar in patients with and without diabetes is in agreement with findings from other high-risk cohorts. [11, 29]

### Potential mechanisms of atrial fibrillation–from cell to serum

AGEs contribute to myocardial remodelling directly via collagen cross-linking and indirectly via interaction with RAGE. [4] Patients with AF have elevated levels of AGEs in atrial tissue and serum compared to those in SR and myocardial AGEs positively correlate with fibrosis. [11, 30] High fat diets promote myocardial AGE and RAGE accumulation, which enhances cardiac inflammation and AGE formation is accelerated by hyperglycaemia and may represent a mechanistic link between diabetes and AF. [16, 31] The receptor for AGEs, RAGE may contribute to tissue repair in response to acute cellular stress, but in chronic disease states such as diabetes and heart failure, excessive ligand accumulation upregulates RAGE expression, perpetuating sustained inflammation and fibrosis—an environment that is conducive to AF. [2–4] Circulating ligands of RAGE are increased in patients with persistent AF compared with paroxysmal AF [10] and SR. [10, 11] RAGE is widely expressed on cardiomyocytes, vascular cells and inflammatory cells and its activation leads to augmented nuclear factor-kappaB activity, which is increased in the atrial tissue of patients with AF compared with SR. [6, 32]

In the present study, the total pool of soluble RAGE and the novel splice variant esRAGE were highest in patients with persistent AF. As esRAGE comprised less than 40% of total soluble RAGE, this would suggest that cleaved RAGE is increased in patients with persistent AF. Both cleaved RAGE and esRAGE are truncated versions of cellular RAGE, capable of binding RAGE ligands without initiating an inflammatory response. The precise relationship between tissue RAGE and serum RAGE levels in man remains unclear, but limited experimental data has shown that serum RAGE levels reflect tissue RAGE levels. [33] It has been suggested that circulating RAGE isoforms may act as decoy molecules by competing with cell-surface RAGE for ligand binding to attenuate vascular damage. [19] However in this study, we speculate that elevated serum levels of sRAGE and esRAGE reflect increased cellular /atrial RAGE, which would amplify inflammation and fibrosis [34] and serve as an enduring substrate for AF maintenance. In agreement with this hypothesis, the majority of circulating sRAGE is generated from membrane cleavage of cellular RAGE, and sRAGE levels positively correlate with AGEs and other inflammatory biomarkers. [35]

Experimental data would also suggest that activation of the AGE/RAGE axis is associated with AF. Diabetic atria exhibit increased arrhythmogenicity and diffuse interstitial fibrosis, which is suppressed by AGE inhibition. [36, 37] Alagebrium (an AGE-inhibitor) reduces myocardial inflammation and collagen cross-linking in association with a reduction in cardiac AGEs and RAGE. [31, 34] Recombinant soluble RAGE also attenuates RAGE-mediated myocardial fibrosis. [38] Angiotensin II receptor blockers reduce both cellular and soluble RAGE, [33] and a meta-analysis reported that renin-angiotensin system blockade prevents incident AF. [39] In a small clinical study of newly identified AF, diabetic patients randomized to pioglitazone showed reduced progression to permanent AF compared with placebo (30% vs. 49%, p = 0.028), in parallel with a reduction in serum AGE levels. [40] Specific anti-RAGE therapies are under development, [41] but have yet to be tested in experimental models of AF or in patients with AF.

### Study limitations

There are study limitations that should be acknowledged. Firstly, AF may be asymptomatic and remain undetected for many years. We may not have captured every patient with paroxysmal AF, although this is unlikely to have impacted on the main study findings. Secondly, the sample sizes for the paroxysmal and persistent AF groups were relatively small, but were adequately powered to detect significant differences in sRAGE and esRAGE. The power to detect a difference with a type 1 error of 5% (two-tailed) was 86% and 85% respectively for the persistent AF versus paroxysmal AF group and 92% and 91% respectively for comparing the persistent AF group with the SR group. Thirdly, this was a cross-sectional study and although we have demonstrated an association between circulating RAGE and persistent AF, direct causation and risk could not be assessed.

## Conclusions

The molecular mechanisms that promote atrial remodeling and maintenance of AF are poorly defined. We report that serum sRAGE and esRAGE are significantly elevated in Caucasian patients with persistent AF. Further clinical studies in larger cohorts are now warranted to establish the potential value of sRAGE levels in predicting the natural history of AF. The therapeutic implications of interrupting the AGE-RAGE axis in patients with AF are unknown, but warrant further investigation.

## Supporting Information

S1 File Serum RAGE levels in the study cohort. ACEi = angiotensin converting enzyme inhibitor; AF = atrial fibrillation; ARB = angiotensin II receptor blocker; CRP = C-reactive protein; eGFR = estimated glomerular filtration rate; RAGE = receptor for advanced glycation end products.
(XLSX)
